# Microstructural Characterization and Prior Particle Boundary (PPB) of PM Nickel-Based Superalloys by Spark Plasma Sintering (SPS)

**DOI:** 10.3390/ma16134664

**Published:** 2023-06-28

**Authors:** Zijun Qin, Qianyi Li, Guowei Wang, Feng Liu

**Affiliations:** The State Key Laboratory of Powder Metallurgy, Central South University, Changsha 410083, China; zijun.qin@csu.edu.cn (Z.Q.); 213311039@csu.cn (Q.L.); wangguowei23@csu.edu.cn (G.W.)

**Keywords:** superalloy, powder metallurgy, spark plasma sintering (SPS), prior particle boundary (PPB)

## Abstract

This research investigates the microstructure and defects of powder metallurgy (PM) nickel-based superalloys prepared by spark plasma sintering (SPS). The densification, microstructural evolution, and precipitate phase evolution processes of FGH96 superalloy after powder heat treatment (PHT) and sintering via SPS are specifically analyzed. Experimental results demonstrate that SPS technology, when applied to sinter at the sub-solidus temperature of the γ’ phase, effectively mitigates the formation of a prior particle boundary (PPB). Based on experimental and computational findings, it has been determined that the presence of elemental segregation and Al_2_O_3_ oxides on the surface of pre-alloyed powders leads to the preferential precipitation of MC-type carbides and Al_2_O_3_ and ZrO_2_ oxides in the sintering necks during the hot consolidation process, resulting in the formation of PPB. This study contributes to the understanding of microstructural modifications achieved through SPS technology, providing crucial information for optimizing sintering conditions and reducing the widespread occurrence of PPB, ultimately enhancing the material performance of PM nickel-based superalloys.

## 1. Introduction

Powder metallurgy (PM) nickel-based superalloys are extensively utilized in gas turbine engines due to their exceptional high-temperature performance [1,2]. The main precipitates of Ni-based superalloys generally are: γ matrix, γ’ precipitation, the topologically close-packed (TCP) phases (σ, μ, P, Laves), and geometrically close-packed (GCP) phases (γ″, η, δ) [3,4]. The preparation process for PM superalloys involves argon atomized (AA)/plasma rotating electrode process (PREP), high-temperature isostatic pressing (HIP), and hot-working. According to the characteristic processes, three main types of defects, namely thermally induced porosity (TIP), non-metallic inclusions, and prior particle boundary (PPB), exist in PM superalloys, with PPB being second-phase particles that nucleate along previous powder edges and cannot be detected by non-destructive testing (NDE) [5]. It is commonly believed that these second-phase constituents, such as carbides, oxides, and carbon oxides, are formed during the HIP process as a result of element segregation. They exist in the form of fine, continuous, or discontinuous particles, which impede the diffusion and bonding of the pre-alloyed powder particles. These constituents act as weak interfaces within the alloy, serving as potential fracture initiators and pathways for crack propagation. Consequently, the presence of these constituents leads to reduced plasticity and diminished overall durability in the resulting alloy [6,7,8,9,10]. Although various methods have been utilized to eliminate PPB, such as large elastic–plastic deformation [11], which results in the breakdown of PPB and fine crystalline particles; powder heat treatment (PHT) of superalloy powders to reduce elemental precipitation on the powder surface; SS-HIP [12], which can reduce the opportunity for the nucleation of second-phase particles along PPB; and microalloy composition, is increasingly popular [10]; for example, hafnium (Hf) can be added to nucleate carbide in the powder. However, none of these methods can fundamentally eliminate the remaining organization of the PPB. Furthermore, the process of interfacial reactions and the mechanism of elimination have not been fully understood. Therefore, further research and discussion are required to investigate the evolution of PPB and the microstructure during HIP of P/M superalloys.

In addition to traditional powder consolidation molding processes such as HIP and hot pressing, a new technology known as spark plasma sintering (SPS) has been proposed and is gaining attention. SPS has the advantages of fast temperature rise, short sintering period, high efficiency, and that fine grains can be obtained [13,14]. In the field of high-temperature alloys, M. Kiani Khouzani et al. [15] studied the microstructure and mechanical properties of SPS cobalt-based superalloy. In the field of cemented carbide, Huang et al. [16] studied the effect of Cu on the densification, microstructures, and properties of WC-6Co under different sintering temperatures by SPS. In the field of intermetallic compound, Zofia et al. [17] investigated the role of the electric current involved in SPS. However, there is little research on the SPS of superalloys, and the sintering mechanism is unclear. Current SPS studies focus on binary and ternary alloy systems or ceramics [18,19,20,21,22], although superalloys consist of more than 10 elements. The effects and mechanism of controlling PPB and grain size by SPS on PM superalloys need to be further investigated. 

Based upon this foundation, the primary objective of this research is to investigate the sintering process systematically and comprehensively and the microstructure of PM nickel-based superalloys prepared by SPS. Moreover, we comprehensively examine the progression of the microstructure evolution, particularly with respect to the PPB, under various electric fields, pressures, temperatures, and times. Furthermore, we explore the mechanisms underlying the observed improvement in the microstructure of the alloy, offering novel insights and approaches for addressing the significant defects in nickel-based high-temperature alloys.

## 2. Materials and Methods

The raw material employed in this study is FGH96 nickel-based superalloy powder produced through argon atomization, and its composition is presented in Table 1. The “Nominal” represents the nominal composition of FGH96 alloy, while the “Actual” indicates the actual elemental composition determined using inductively coupled plasma-atomic emission spectrometry (ICP-AES). The D(v,0.5) is 30.47 μm.

**Figure 1:**
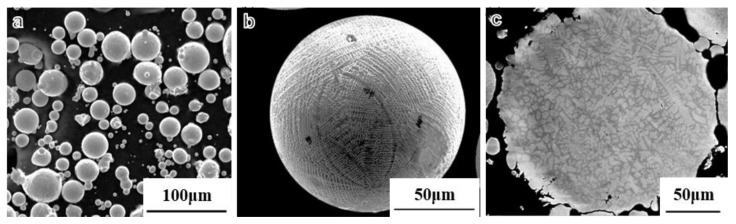
The microstructure of superalloy powders (**a**) The surface microstructure of superalloy powders (**b**) The surface microstructure of a superalloy powder (**c**) The section microstructure of a superalloy powder.

Figure 1 displays the SEM microstructure images of the surface and cross-section of the alloy powders. In Figure 1a, it can be observed that the powders exhibit a predominantly spherical shape. However, there are also a small amount of satellite-like agglomerated powders, encapsulated powders, fragmented powders, and irregular powders. The particle size distribution is broad, with most powder diameters being smaller than 50 μm, and even containing numerous powders smaller than 10 μm. Figure 1b presents an enlarged view of an individual powder surface, revealing that the microstructure on the particle surface is primarily composed of dendritic and cellular structures. Figure 1c displays an enlarged view of an individual powder cross-section, showing a consistent microstructure with the surface, primarily comprising dendritic and cellular structures.

The processing method and parameters for each sample are presented in Table 2. To compare the samples prepared by SPS, a PHT experiment was conducted. The SPS equipment used was the FCT D25/3 from Germany. Each sample weighed approximately 18 g and was wrapped with a layer of graphite paper before being loaded into cylindrical dies with an inner diameter of 20 mm. The pulse pattern employed was 12-2, consisting of 12 pulses followed by 2 rest intervals. A heating rate of 100 °C/min was applied to reach the final temperature. The environment pressure during the process was maintained at 50 MP. For cooling, circulating water in the stove was used. Considering that the solvus temperature of γ’ phase, as calculated by Thermo-Calc software (version 3.0.1), is approximately 1120 °C (as stated in Section 4.1 of this study), two different compaction temperatures, 1070 °C and 1170 °C, were chosen to observe the variations in alloy consolidation and microstructure under the above-solvus and sub-solvus temperatures. Additionally, two different holding times were employed at the desired temperature: 5 min and 40 min. In summary, a total of eight different samples were obtained in this study. The naming convention for the samples is as follows: the first position represents the sample type, with S/P denoting SPS samples and powder samples, respectively. The second position represents the sintering/heat treatment temperature, with L/H indicating low temperature (1070 °C) and high temperature (1170 °C), respectively. The third position corresponds to the sample number, with 1/2 representing a sintering time of 5 min or 40 min, respectively. 

The sintered samples were sectioned in half and subsequently embedded in resin to expose their interior for mechanical grinding and polishing. Microstructural analysis was conducted using optical microscopy (OM, Leica DM4000M, Wetzlar and Mannheim, Germany), scanning electron microscopy (SEM, FEI Quanta 650 FEG, Hillsboro, OR, USA), electron probe X-ray micro-analyzer (EPMA, JEOL JXA-8530F, Tokyo, Japan) and electron backscattered diffraction (EBSD, OXFORD NordlysMax, Oxford, UK). The polished surface was etched using 15 mL HCl, 10 mL acetic acid, and 10 mL HNO3 for optical observation.

## 3. Results

### 3.1. The Densification Process and Microstructure of SPS Samples

**Figure 2:**
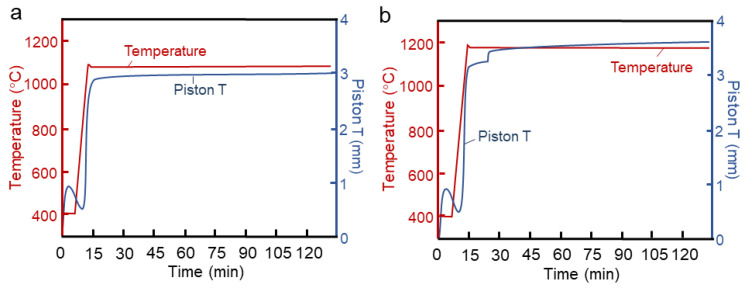
The sintering curves of materials (**a**) 1070 °C/120 min (**b**) 1170 °C/120 min.

To investigate the sintering process of the alloy, we conducted sintering experiments for over 120 min, and the sintering curve is shown in Figure 2. The red line represents the temperature variation during sintering, while the blue line represents the displacement of the sample shrinkage probe as detected by the equipment. Combining the experimental process and the sintering curve, it can be observed that when the displacement curve of the probe reaches its first peak, the main reason for the alloy densification is the pressing pressure from the mold, which compacts the initially loosely packed powder. Subsequently, as the sintering temperature continues to rise, the displacement curve of the probe exhibits significant fluctuations. The alloy undergoes expansion followed by rapid densification, primarily due to the solid-state sintering process, which involves particle bonding, densification, and complex phase transformations that occur at high temperatures.

At a sintering temperature of 1070 °C (Figure 2a), the sample completes the densification process after approximately 20 min of sintering. Subsequent sintering has minimal impact on the alloy densification. At a sintering temperature of 1170 °C (Figure 2b), a plateau region appears in the densification process of the alloy after approximately 20 min of sintering. The density of the alloy increases again around 26 min, suggesting a further phase transformation behavior at high temperatures. Thus, the alloy essentially completes the densification process after a sintering hold time of 40 min, and the impact of sintering beyond 40 min on alloy densification is minimal, primarily involving grain growth processes.

**Figure 3:**
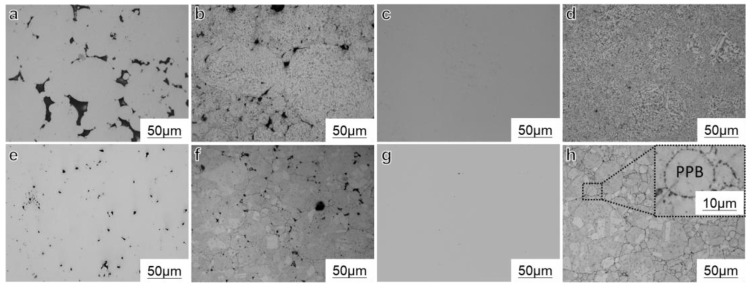
The microstructure of (**a**) SL1, (**c**) SL2, (**e**) SH1, (**g**) SH2, and (**b**,**d**,**f**,**h**) the alloys after etching were observed by optical microscope.

The microstructure of the samples prepared by SPS are depicted in Figure 3. In order to better observe the densification process of the alloy during the SPS process, Figure 3a,c,e,g show the surface microstructure of the unetched samples, while the remaining figures display the microstructure after etching. From the images, it can be observed that a significant number of pores with various shapes and sizes were observed in samples SL1 and SH1, indicating incomplete compaction of the alloy. Conversely, samples SL2 and SH2 exhibited no visible pores, indicating successful densification of the alloy. As the sintering temperature increased, the porosity of the alloy decreased significantly at the same sintering time.

The microstructure of the samples prepared at 1070 °C exhibited a peculiar pattern. Typically, equiaxed grains are observed after sintering using methods such as hot isostatic pressing (HIP) or SPS at 1170 °C. However, when the sintering temperature was set at 1070 °C, the microstructure consisted of cellular and dendritic crystals in samples SL1 and SL2. Despite its similarity to the microstructure of the pre-alloyed powder, a comparison with Figure 1 reveals that the lengths of the cellular and dendritic crystals in pre-alloyed powder typically range from 5–50 μm. In contrast, the SL2 sample exhibits significantly smaller crystals, with some even less than 1 μm, indicating a finer microstructure compared to the pre-alloyed powder. Notably, distinct sintered necks were formed between the powder particles, and their growth was evident. In addition to neck growth, a significant plastic deformation process occurred in the alloy powder. Under forming pressure, the powder particles were compressed, leading to severe plastic deformation and the formation of sintered necks with surrounding powders. Surprisingly, the presence of PPB was not prominent.

In contrast, the alloy sintered at higher temperatures exhibited almost no discernible powder morphology, and equiaxed crystals were formed. Notably, the PPBs were clearly evident in the fully dense SH2 sample. The precipitated phases on the PPBs maintained the original spherical distribution characteristics of the powder, suggesting that the densification mechanism of the alloy involved solid-phase sintering, with less plastic deformation of the powder particles occurring.

Density measurements were performed using the Archimedes method and compared with the density values of the master alloy and the alloys prepared by the 1170 °C/120 min HIP process from the same batch of powders, as shown in Table 3. The density data for each experimental condition were obtained by averaging measurements from more than five samples. From the table, it can be observed that, except for the SL2 sample, the densities of the alloys prepared by the SPS process are lower than those of the master alloy and the HIPed alloys. Considering the microstructure images, it can be inferred that both the SL2 and SH2 samples have undergone sintering and achieved densification. Therefore, the main influence on the alloy density should be attributed to the different phase structures within the alloy.

### 3.2. The Effect of Temperature and Technology on Grain Size

**Figure 4:**
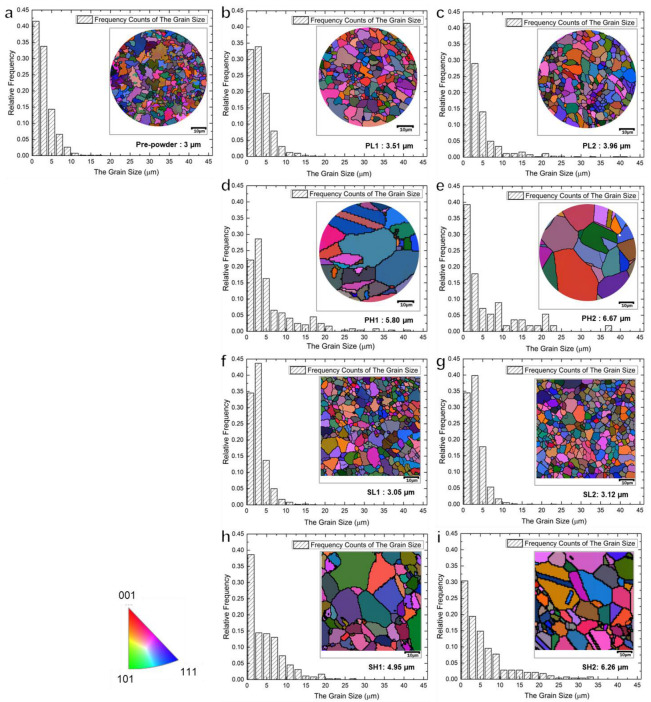
The grain size and orientation of samples (**a**) pre-powder (**b**) PL1 (**c**) PL2 (**d**) PH1 (**e**) PH2 (**f**) SL1 (**g**) SL2 (**h**) SH1 (**i**) SH2.

EBSD analysis was conducted to investigate the grain orientation and grain size distribution characteristics of alloys in different states, including the pre-alloyed powder, powder samples (PL1, PL2, PH1, and PH2), and bulk samples (SL1, SL2, SH1, and SH2), as shown in Figure 4. The grain size distribution histogram was plotted based on EBSD analysis statistics of at least three different areas, defining grains with an orientation difference greater than 15° as separate entities. The left side of each histogram represents the grain orientation distribution of the corresponding sample, with different colors indicating different grain orientations. The specific grain orientation reference can be found in the bottom left corner of the orientation standard color chart. The average grain size of each sample is shown in the bottom right corner of the respective image. 

The results indicate that all samples did not show any preferred grain orientation throughout the entire processing sequence. Additionally, the average grain size of the pre-powders was 3 μm. The average grain sizes of PL1, PL2, SL1, and SL2 samples processed at 1070 °C were all below 4 μm. However, the PH1, PH2, SH1, and SH2 samples processed at 1170 °C exhibited varying degrees of grain growth, ranging from 4.9 μm to 6.7 μm, with the values increasing with prolonged holding time. It is worth noting that the average grain sizes of the SPS samples were smaller compared to the corresponding powder samples under the same conditions. This suggests that sintering at sub-solidus temperatures can effectively control grain growth in the alloy, and the SPS method is capable of inhibiting grain growth effectively.

In addition to the differences in grain size, twins were observed in PH1, PH2, and SH2, which were sintered at higher temperatures. Due to their low stacking fault energy, nickel-based superalloys are prone to twin formation during recrystallization. The recrystallization driving force of the pre-alloys was lower at lower sintering temperatures, resulting in recrystallization occurring in the later stages of sintering. Conversely, at higher sintering temperatures, the recrystallization driving force of the alloy increased, leading to a faster recrystallization process. Consequently, no twins were observed at a sintering temperature of 1070 °C, while evident twins were observed at a sintering temperature of 1170 °C.

### 3.3. The Precipitations under Different Processing Conditions

**Figure 5:**
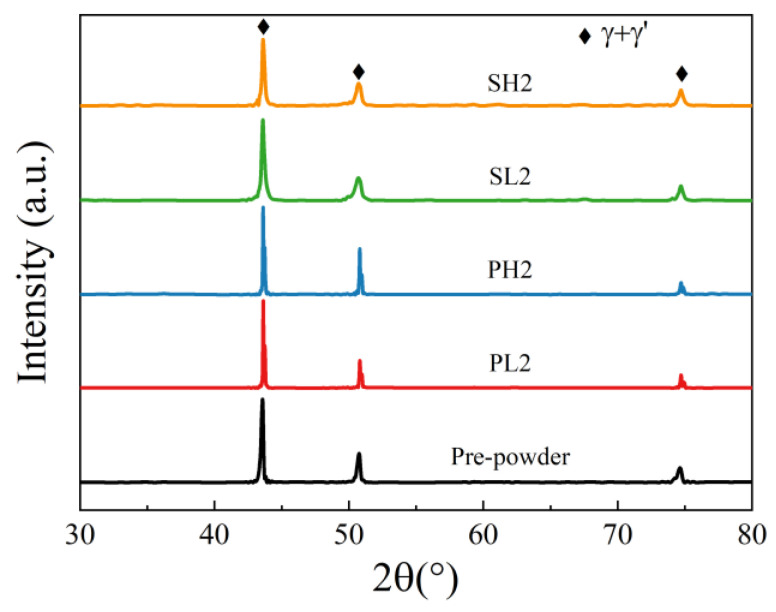
XRD patterns of pre-powder, PL2, PH2, SL2, and SH2 samples.

To analyze the precipitate phases in the alloy, X-ray diffraction (XRD) characterization was performed on the pre-powder, PL2, PH2, SL2, and SH2 samples, as shown in Figure 5. From the XRD patterns, it is evident that the major phases in all samples are the γ matrix and the γ’ strengthening phases. It should be noted that XRD can only characterize precipitate phases with a content greater than 5% in the alloy. Since the content of carbon oxides and other second phases in the alloy are minimal, further characterization of the other precipitate phases in the alloy, as well as exploration of the elemental segregation phenomena, was conducted using EPMA on the five samples.

**Figure 6:**
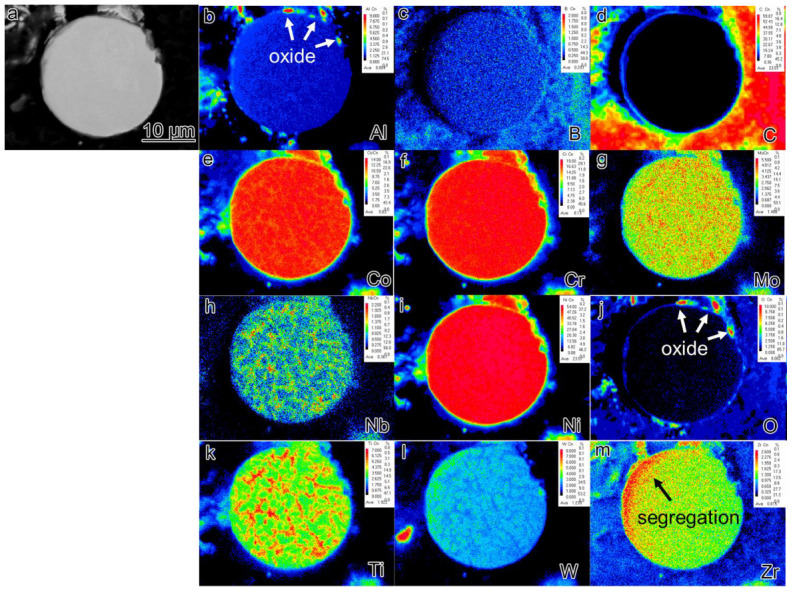
The element segregation of pre-alloyed powder (**a**) BSE image of collecting places by EPMA (**b**–**m**) corresponding elements (Al, B, C, Co, Cr, Mo, Nb, Ni, O, Ti, W, Zr) mapping.

**Figure 7:**
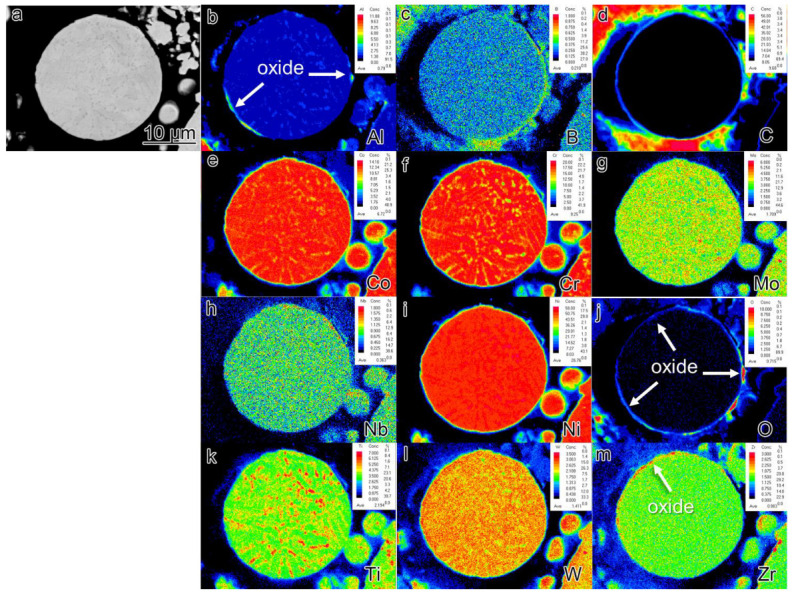
The element segregation of PL2 (**a**) BSE image of collecting places by EPMA (**b**–**m**) corresponding elements (Al, B, C, Co, Cr, Mo, Nb, Ni, O, Ti, W, Zr) mapping.

**Figure 8:**
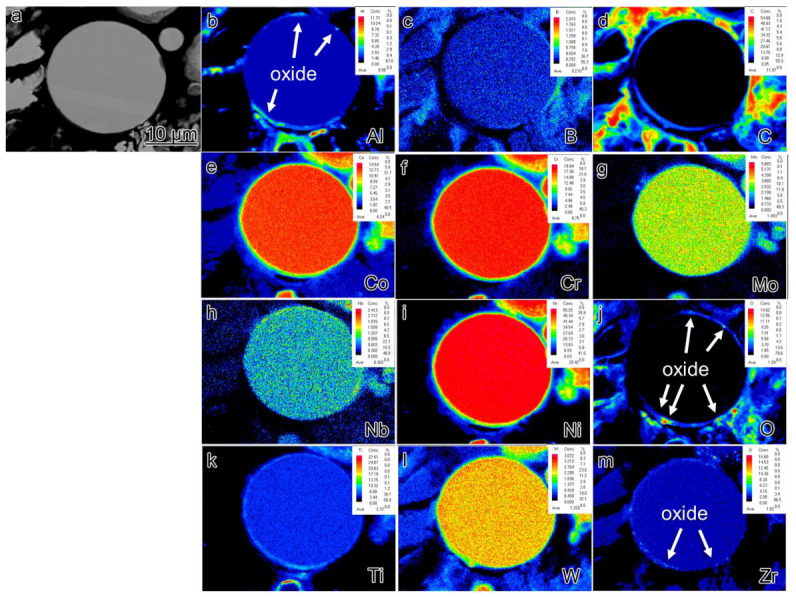
The element segregation of PH2 (**a**) BSE image of collecting places by EPMA (**b**–**m**) corresponding elements (Al, B, C, Co, Cr, Mo, Nb, Ni, O, Ti, W, Zr) mapping.

EPMA analysis was conducted on the sections of pre-alloyed powder and powder after heat treatment (PHT); the results are presented in Figure 6, Figure 7 and Figure 8. The upper right corner of each map indicates the corresponding element content, with higher element content indicated by a shift towards a reddish color in the corresponding regions of the map, while lower element content is represented by a bluish color. The powder samples, with a selected diameter of approximately 25–30 μm, were embedded in resin for analysis. It can be observed that element segregation was present in all powder samples, and with increasing temperature, the distribution of alloy elements became more uniform.

In the pre-alloyed powder, the Co and W elements exhibited segregation within the cellular crystals, while the Mo, Nb, and Ti elements showed segregation at the boundaries of the cellular crystals. Additionally, the Zr element was observed to be segregated on the surface of the alloy powder. In PH2, the Co, Cr, W, and Mo elements exhibited segregation within the cellular crystals, while the Nb, Ti, Al, and Ni elements displayed segregation at the boundaries of the cellular crystals. Furthermore, small, precipitated phases enriched with Mo, Nb, and Ti were observed internally, and an enriched layer of Zr was present on the powder surface. The heat treatment induced element diffusion within the powder alloy, with the constituent elements of the γ’ phase gradually migrating and forming small γ’ precipitates at the boundaries of the cellular crystals. The internal element distribution of PH2 was more uniform. Since the heat treatment temperature exceeded the γ’ solution temperature, the primary γ’ phase in the alloy was completely dissolved, and during the cooling process, a large number of fine secondary γ’ phases were formed. Additionally, the segregation of Nb, Ti, and Zr on the powder surface indicated the presence of small Nb, Ti, and Zr-rich precipitates on the powder surface.

**Figure 9:**
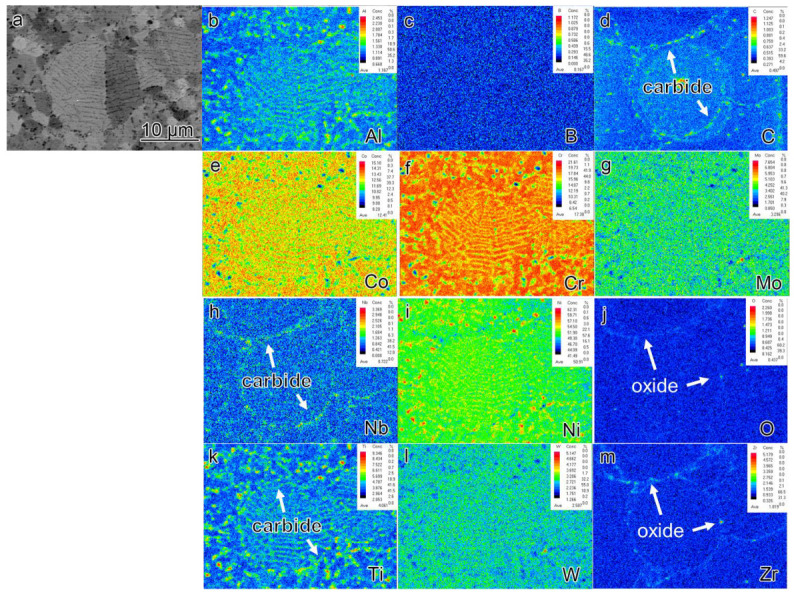
The element segregation of SL2 (**a**) BSE image of collecting places by EPMA (**b**–**m**) corresponding elements (Al, B, C, Co, Cr, Mo, Nb, Ni, O, Ti, W, Zr).

**Figure 10:**
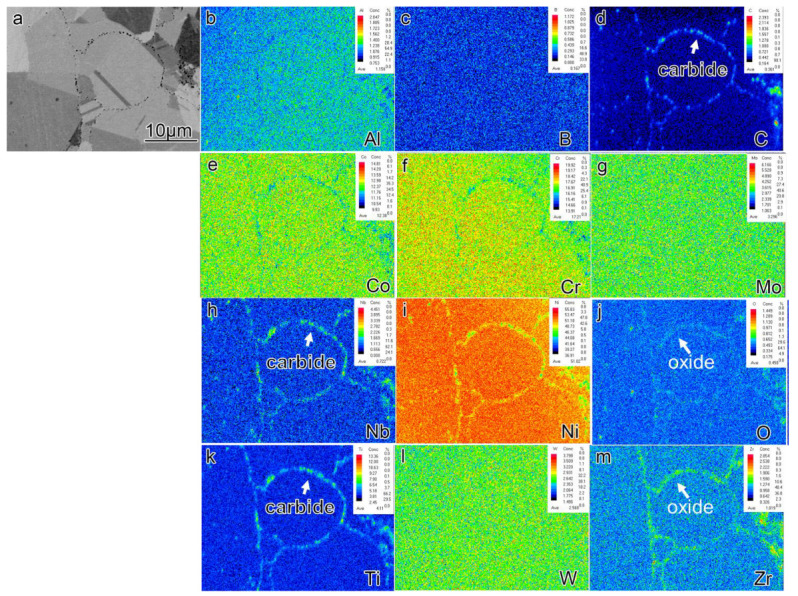
The element segregation of SH2 (**a**) BSE image of collecting places by EPMA (**b**–**m**) corresponding elements (Al, B, C, Co, Cr, Mo, Nb, Ni, O, Ti, W, Zr).

Figure 9 and Figure 10 depict the EPMA images of SL2 and SH2, respectively. It should be noted that, as shown in Figure 3, the overall presence of PPB in the SL2 sample is not prominent. In order to observe the PPB in the alloy, we deliberately sought out a region with more noticeable defects for observation. The application of electric field and pressure during the SPS method resulted in a more pronounced segregation of γ’ constituent elements compared to the powders after PHT at 1070 °C. Additionally, a greater amount of nanoscale oxycarbide phases was observed in the samples prepared by SPS. Comparing Figure 7 and Figure 8 reveals that the surface of the powder samples exhibits only a few instances of continuous or discontinuous oxide segregation, while the bulk sample contains a significant amount of carbides, with carbon oxides precipitating almost continuously along the PPB, forming a thin film. The elements forming carbon oxides are mainly C, O, Nb, Ti, and Zr. Notably, even in the region of the SL2 sample where the precipitation of PPB is most severe, the amount of carbon oxides precipitated is significantly lower compared to the SH2 sample. This indicates that the sintering temperature of SPS has a significant influence on the precipitation behavior of PPB.

## 4. Discussion

### 4.1. The Thermodynamic and Kinetic Calculation 

**Figure 11:**
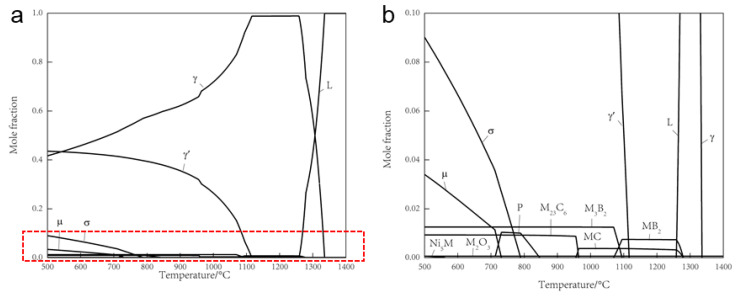
(**a**) The equilibrium phase diagram alloy calculated by Thermo-Calc software (**b**) Zoomed-in view of the highlighted region in Figure (**a**).

Figure 11 illustrates the thermodynamic equilibrium phase diagram calculated using Thermo-Calc with the TTNi8 database. The solidus temperature of the alloy is determined to be 1258 °C, the liquidus temperature is 1336 °C, and the γ’ complete solution temperature is 1116 °C. The higher solid–liquid phase boundary of the alloy is primarily attributed to the significant presence of high-melting-point elements, such as W, Mo, Nb, Hf, in the alloy composition. The incorporation of these elements not only elevates the melting point of the alloy but also enhances its high-temperature strength.

The calculated possible precipitated phases in the alloy include Ni_3_M, M_2_O_3_, M_23_C_6_, M_3_B_2_, MC, MB_2_, μ phase, σ phase, and P phase. The types and contents of precipitates are presented in Table 4 and Table 5. The predominant precipitates at both temperatures were identified as MC carbide, accompanied by a small quantity of M_2_O_3_ oxide, which is consistent with the experimental detection. However, the presence of boride precipitates at 1070 °C was not detectable via EPMA due to the light weight and low content of boron atoms.

Based on thermodynamic calculations and experimental results, the precipitate phases observed in the alloy are primarily γ’, ZrO_2_, and M_2_O_3_ oxide rich in Al and Ti. Additionally, there are MC carbides rich in Ti and Nb.

The formation of PPB can be traced back to the pre-alloyed powder. Figure 6 reveals that element segregation occurs on the surface of the pre-alloyed powder after rapid cooling and is also observed within the dendrite–interdendrite and cellular–intercellular. The dendrite–interdendrite and cellular–intercellular element segregation primarily occurs due to the different undercooling levels between the γ matrix and the γ’ strengthening phase. During the formation of pre-alloyed powder, the γ matrix solidifies first as dendrites or cellular structures, leading to the preferential segregation of constituent and solution elements, such as Co, Cr, W, and Mo. Subsequently, γ’ forming/solution elements such as Nb, Ti, and Al solidify within the intercellular and interdendrite. However, the mechanism behind the formation of the segregated layer consisting of Al_2_O_3_ oxide and Zr elements on the powder surface remains unclear. Furthermore, a significantly higher quantity of precipitates is observed in the PHT and SPS samples compared to the pre-alloyed powder. In the bulk alloy, not only Al_2_O_3_ but also ZrO_2_ oxide is present. 

Burachynsky et al. [23] and Swalin et al. [24] have calculated diffusion coefficients for several elements in Ni. They observed that the diffusion coefficient of solute atoms in Ni is negatively correlated with the atomic radius of the solute, meaning that elements with larger atomic radii have smaller diffusion coefficients. The atomic size sequence of all added elements in our research investigated is as follows: Ni < Co < Cr < Mo < W < Al ≈ Nb < Ta < Ti < Hf < Zr. This indicates a higher tendency for the diffusion and precipitation of carbon oxides composed of elements such as Zr, Hf, Ti, Ta, and Nb. 

Notably, Al_2_O_3_ has a standard molar enthalpy of formation of −1675.3 KJ/mol, ZrO_2_ has a standard molar enthalpy of formation of −1097.5 KJ/mol, and the Ni3Al has a standard molar enthalpy of formation of −282.4 KJ/mol [25]. Combining this information with Table 5, it is apparent that Al has a strong tendency to form oxides on the alloy powder surface. Moreover, at elevated temperatures, Zr could potentially undergo a displacement reaction with Al_2_O_3_, where the change in reaction free energy is negative [26]:

(1) $$2{Al}_{2}O_{3}+3Zr+12Ni\overset{1300K}{\to }3ZrO_{2}+4{Ni}_{3}Al\;∆G=-618.4KJ/mol$$

During the sintering process, Al_2_O_3_ within the powder is replaced by Zr, leading to the formation of ZrO_2_ and γ’ phase. Simultaneously, the Al_2_O_3_ present on the surface of the pre-alloyed powder particles also undergoes Zr substitution. As a result, no precipitated Al_2_O_3_ phase is observed in the sintered alloy, but the presence of ZrO_2_ precipitates is detected.

In order to further explore the formation mechanism of carbon oxides, we have incorporated the study of elemental diffusion in alloys conducted by Luthra [27]. The thermodynamic calculation model could be used to calculate the segregation ratio of the interior and surface of different phases $X_{B}^{s}/X_{B}^{b}$:

(2) $$\frac{X_{B}^{s}}{X_{B}^{b}}=exp[\frac{∆G_{{AB}_{x}}^{o}\left(1-f_{1}\right)}{xRT}]\cdot exp[\frac{{\sigma f_{2}\Gamma }_{B}^{o}}{RT}]\cdot {\left[\frac{1}{X_{B}^{b,1}\cdot {\left(a_{B}^{b,1}\right)}^{1/x}}\right]}^{\left(1-f_{1}\right)}$$

where $X_{B}^{s}$ represented the volume fraction of component B in the bulk phase, $X_{B}^{s}$ represented the volume fraction of component B on the surface, $∆G_{{AB}_{x}}^{o}$ represented the standard free energy of formation of ${AB}_{x}$, R is the molar gas constant, T is the thermodynamic temperature. σ is the surface tension, $\Gamma_{B}^{o}$ represented the single-layer spread area of pure material B, $X_{B}^{b,1}$ represented the solubility limit of component B in the bulk phase, and $a_{B}^{b,1}$ represented the activity of dominating metal elements in the bulk. The $f_{1}$ and $f_{2}$ are coefficients, depending on the lattice coefficient of the alloy and the crystal structure of the segregation phase.

The segregation ratio $X_{B}^{s}/X_{B}^{b}$ of the MC-type carbide and M_2_O_3_-type oxide in the interior and surface of the matrix in the nickel-based superalloy could be calculated. According to the existing research [28,29,30,31], they approximated the matrix as pure nickel, so:

(3) $${\sigma \Gamma_{i}^{o}=\mu }_{i}^{o,s}-\mu_{i}^{o,b}=117.5KJ/mol$$

where $\mu_{i}^{o}$ is the chemical potential of the pure material, and $f_{1}=0.76$, $f_{2}=1$. The calculation results of the mainly MC-type carbides and M_2_O_3_-type oxide are shown in Table 6. The computational results indicate a strong tendency for C to accumulate on the powder surface at high temperatures, leading to the formation of TiC and NbC carbides. Therefore, carbides have a propensity to precipitate at PPB during the thermal consolidation process. In contrast, the precipitation tendency for Al_2_O_3_ oxide during the thermal consolidation process is weaker compared to that of carbides. Therefore, the observed precipitation phase in the PPB predominantly consists of a significant amount of carbides with a minimal presence of oxides.

### 4.2. The Evolution of PPB

**Figure 12:**
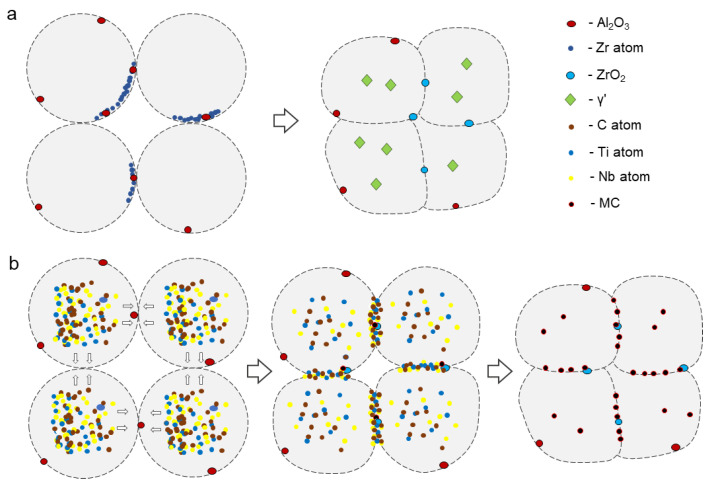
The evolution of PPB. (**a**) oxides (**b**) carbides.

Based on the comprehensive thermodynamic calculations and experimental results, it can be concluded that the formation mechanism of the PPB is primarily attributed to the formation processes of carbides and oxides. Figure 12a depicts the formation process of oxides during sintering. As a result of the existing segregation of Al_2_O_3_ and Zr elements on the surface of the pre-alloyed powder, Al_2_O_3_ is displaced by the Zr element, leading to the formation of ZrO_2_ and γ’ phase under high temperature and pressure during the sintering process.

Figure 12b illustrates the formation process of carbides during sintering. The sintering process facilitates the segregation of carbide composition elements such as C, Ti, and Nb due to their high diffusion coefficients. These elements tend to segregate at the sintered neck. Owing to the lower nucleation energy of carbides [20,34] compared to oxides at the interface, MC type carbides precipitate in the sintered neck.

Despite the less apparent presence of PPB in PL2, a certain amount of oxycarbide was observed within the matrix. This can be attributed to the sintering mechanism, where the pre-alloyed powder experienced plastic deformation prior to solid-phase sintering. As a result, the particle boundaries of the powder were partially disrupted. Since the sintering temperature was relatively low, the formation of the sintering neck occurred gradually. Consequently, the diffusion of carbide-forming elements towards the powder boundaries was incomplete, leading to nucleation within the matrix.

## 5. Conclusions

Based on the afore mentioned results and discussions, the following conclusions can be drawn:

(1) During the rapid solidification process, there were very few Al_2_O_3_ precipitates on the surface of the pre-alloyed powders. Subsequently, after heat treatment, Nb and Ti-rich carbides are observed in the powders.
(2) The SPS samples exhibited almost negligible PPB at a sintering temperature of 1070 °C. Conversely, the presence of prominent PPB was observed at a sintering temperature of 1170 °C. This indicates that the SPS method is effective in reducing PPB formation at low sintering temperatures while facilitating grain size control within a short duration.
(3) The mechanism underlying PPB formation involves the segregation of certain elements already present on the surface of the pre-alloyed powder, with carbides and oxides preferentially precipitating at the sintering neck under the influence of thermodynamic and dynamic factors. The predominant precipitates within the PPB are ZrO2 and MC carbides enriched with Ti and Nb elements.

## Figures and Tables

**Table 1:** The composition of powders.

	Co	Cr	Mo	W	Al	Ti	Nb	B	Zr	C	Ni
Nominal	13	16	4	4	2.3	3.75	0.90	0.013	0.04	0.05	Bal.
Actual	12.36	16.68	3.75	3.89	2.18	3.95	1.05	0.094	0.036	0.0416	Bal.

**Table 2:** The processing method and parameters of each simple.

Parameters	SL1	SL2	SH1	SH2	PL1	PL2	PH1	PH2
Method	SPS	SPS	SPS	SPS	PHT	PHT	PHT	PHT
Temperature (°C)	1070	1070	1170	1170	1070	1070	1170	1170
Time (min)	5	40	5	40	5	40	5	40

**Table 3:** The density of alloys prepared by different deposition methods.

Preparation Process	SL1	SL2	SH1	SH2	Master Alloy	After-HIPed
Density (g/cm^3^)	8.1291	8.3104	8.2543	8.2608	8.2937	8.2931

**Table 4:** The precipitated phases and content in alloy at 1070 °C and 1170 °C.

Temperature (°C)	Mole Fraction/%					
	γ	MB_2_	M_2_O_3_	γ’	M_3_B_2_	MC
1070	83.26	0.02	0.06	15.07	1.22	0.37
1170	98.84	0.74	0.06	-	-	0.36

**Table 5:** The content of MC-type carbide and M_2_O_3_-type oxide.

Temperature (°C)	Phase	Composition Mole Fraction/%									
		C	Ti	Zr	Nb	W	Mo	Cr	O	Al	Ti
1070	MC	47.65	38.31	0.81	11.55	1.16	0.20	0.31	-	-	-
	M_2_O_3_	-	-	-	-	-	-	-	60.00	39.81	0.19
1170	MC	47.48	40.37	0.48	9.84	1.23	0.25	0.34	-	-	-
	M_2_O_3_	-	-	-	-	-	-	-	60.00	39.62	0.38

**Table 6:** Segregation at 1100 °C.

Compound	Dominating Metal	$∆G_{MC}^{o}$ (kJ/mol)	$a_{B}^{b,1}$	$X_{B}^{b,1}$	$X_{B}^{s}/X_{B}^{b}$
TiC	Ti	−168.4 [28]	2.59 × 10^−4^ [30]	5 × 10^−4^	15,252
NbC	Nb	−127.8 [28]	4.5 × 10^−5^ [31]	5 × 10^−4^	35,813
Al_2_O_3_	Al	−1249.5 [32]	3.52 × 10^−8^ [27]	6.6 × 10^−4^ [33]	200.7

## Data Availability

The data supporting the findings of this work are available in the main text. Raw data are available from the corresponding authors on reasonable request.
